# Synthesis and Structural Characterization of a Series of One-Dimensional Heteronuclear Dirhodium-Silver Coordination Polymers

**DOI:** 10.3390/polym11010111

**Published:** 2019-01-10

**Authors:** Paula Cruz, Estefania Fernandez-Bartolome, Miguel Cortijo, Patricia Delgado-Martínez, Rodrigo González-Prieto, José L. Priego, M. Rosario Torres, Reyes Jiménez-Aparicio

**Affiliations:** 1 Departamento de Química Inorgánica, Facultad de Ciencias Químicas, Universidad Complutense de Madrid, Ciudad Universitaria, E-28040 Madrid, Spain; paula.cruz@urjc.es (P.C.); estefania.fernandez@imdea.org (E.F.-B.); rodgonza@ucm.es (R.G.-P.); 2 Centro de Asistencia a la Investigación Difracción de Rayos X, Facultad de Ciencias Químicas, Universidad Complutense de Madrid, E-28040 Madrid, Spain; patriciadelgado@ucm.es (P.D.-M.); mrtorres@quim.ucm.es (M.R.T.)

**Keywords:** dirhodium compounds, dicyano-argentate complexes, heteronuclear, one-dimensional, rhodium-silver chains, phenyl embrace

## Abstract

Herein, we describe the preparation of heteronuclear dirhodium-silver complexes by reaction between molecular Rh(II)-Rh(II) compounds [Rh_2_(μ-O_2_CR)_4_L_2_] (R = Me, Ph (**1**), CH_2_OEt (**2**); L = solvent molecules) with paddlewheel structure and PPh_4_[Ag(CN)_2_]. One-dimensional coordination polymers of (PPh_4_)_n_[Rh_2_(μ-O_2_CR)_4_Ag(CN)_2_]_n_ (R = Me (**3**), Ph (**4**), CH_2_OEt (**5**)) formula have been obtained by replacement of the two labile molecules in the axial positions of the paddlewheel structures by a [Ag(CN)_2_]^−^ bridging unit. The crystal structures of **3**–**5** display a similar arrangement, having anionic chains with a wavy structure and bulky (PPh_4_)^+^ cations placed between the chains. The presence of the (PPh_4_)^+^ cations hinders the existence of intermolecular Ag-Ag interactions although several C-H····π interactions have been observed. A similar reaction between [Rh_2_(μ-O_2_CCMe_3_)_4_(HO_2_CCMe_3_)_2_] and PPh_4_[Ag(CN)_2_] led to the molecular compound (PPh_4_)_2_{Rh_2_(μ-O_2_CCMe_3_)_4_[Ag(CN)_2_]_2_} (**6**) by replacement of the axial HO_2_CCMe_3_ ligands by two [Ag(CN)_2_]^−^ units. The trimethylacetate ligand increases the solubility of the complex during the crystallization favouring the formation of discrete heteronuclear species.

## 1. Introduction

**Figure 1:**
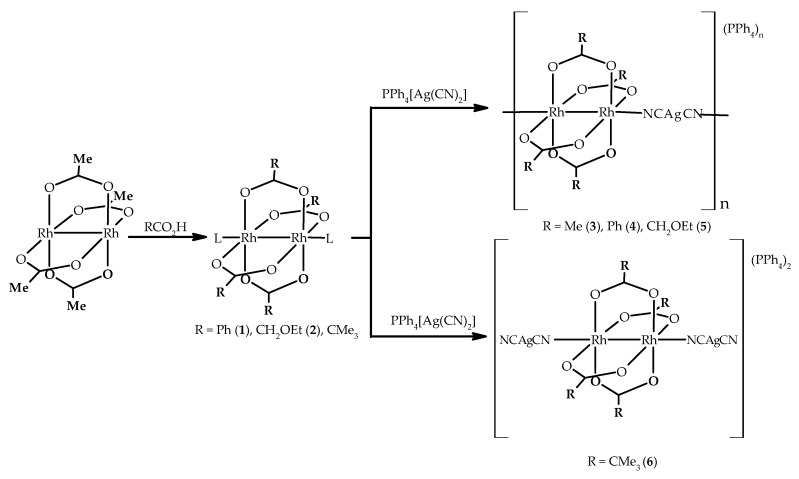
Synthetic approach employed to prepare **1**–**6**.

In the chemistry of compounds with a direct metal-metal bond the dirhodium complexes play a prominent role because their properties span very different fields [1,2,3]. Many of the Rh_2_ complexes adopt the paddlewheel structure shown in Figure 1. The majority of these compounds contain a diamagnetic Rh_2_^4+^ core with a σ^2^π^4^δ^2^δ*^2^π*^4^ electronic configuration leading to a net metal-metal bond order of one. Although dirhodium compounds have been extensively studied due to their potential applications in several fields such as catalysis [4,5,6,7] or biological or antitumoral activity [8,9,10,11,12], the variety of architectures in these type of complexes have also attracted a huge interest [13,14,15,16,17,18,19].

As the axial positions of the [Rh_2_(μ-O_2_CR)_4_] (R = alkyl or aryl) are usually occupied, a great amount of molecular complexes of the type [Rh_2_(μ-O_2_CR)_4_L_x_] (L = monodentate donor ligand; x = 1, 2) have been described [1,2,3,20,21,22]. Nevertheless, the Rh_2_^4+^ unit presents in the tetracarboxylatodirhodium(II) complexes has been used to achieve a large number of coordination polymers. One-dimensional polymers have also been obtained using bridging ligands between the dirhodium units [16,17,18,19].

Heterometallic one-dimensional compounds containing M_2_ units with direct metal-metal bonds and other transition metal complex are very useful as functional materials due to their versatile physical and chemical properties [23,24]. A very useful strategy to form heterometallic complexes has been developed by Petrukhina et al. [25] using the electrophilic tetrakis(trifluoroacetato)dirhodium(II) complex, [Rh_2_(μ-O_2_CCF_3_)_4_]. Thus, the reaction of [Rh_2_(μ-O_2_CCF_3_)_4_OCMe_2_]_2_ with the tetranuclear species [Cu_4_(O_2_CCF_3_)_4_] afforded a bidimensional coordination polymer of {[Rh_2_(μ-O_2_CCF_3_)_4_]·μ_2_-OCMe_2_·[Cu_4_(O_2_CCF_3_)_4_]} formula. In addition, several heterometallic Rh-Pt one-dimensional complexes have been described [26,27,28,29,30]. For example, {[Rh_2_]−[Pt_2_] −[Pt_2_]}_n_ or [Rh_2_]−[Pt−Cu−Pt] polymers with dimetallic units joined by unbridged Pt-Rh metal-metal bonds have been obtained [26,27,28,29,30,31]. Moreover, a strategy to construct one-dimensional compounds from dirhodiumtetracarboxylato units, [Rh_2_(μ-O_2_CR)_4_]^+^ (R = Me, Et) using dicyanoaurate, [Au(CN)_2_]^−^ groups as linker has been developed by our research group [32].

The negative charge of anionic one-dimensional chains based on dirhodium compounds is, usually, compensated by alkali cations [32]. However, the use of a non-innocent counterion as tetraphenylphosphonium could give other possibilities of arrangement. Thus, it is known that in compounds containing tetraphenylphosphonium or triphenylphosphane groups the phenyl-phenyl interactions can combine to give several attractive supramolecular motifs. For example sextuple, quadruple, or double phenyl embraces have been observed [33,34,35,36]. This multiple phenyl embrace can determine the arrangement of the compound in the solid state and is very important in the develop and design of supramolecular structures [37,38,39].

Following the antecedents described above, we have prepared dirhodium-silver anionic one-dimensional coordination polymers of (PPh_4_)_n_[Rh_2_(μ-O_2_CR)_4_Ag(CN)_2_]_n_ (R = Me (**3**), Ph (**4**), CH_2_OEt (**5**)) formula by reaction between discrete dinuclear building blocks with two labile ligands in the axial positions, [Rh_2_(μ-O_2_CR)_4_L_2_] (R = Me, Ph (**1**), CH_2_OEt (**2**); L = solvent molecules) and PPh_4_[Ag(CN)_2_] (Figure 1). The same approach has also lead to a molecular compound of (PPh_4_)_2_{Rh_2_(μ-O_2_CCMe_3_)_4_[Ag(CN)_2_]_2_} (**6**) formula. The presence of intermolecular interactions including multiple phenyl embraces between the phenyl rings of the PPh_4_^+^ counterion have also been examined.

## 2. Materials and Methods

### 2.1. Materials

PPh_4_[Ag(CN)_2_] was prepared by mixing a solution of 0.2 mmol (0.04 g) of K[Ag(CN)_2_] in 4 mL of water with a solution of 0.2 mmol (0.08 g) of PPh_4_Br in 8 mL of water and stirring for 5 min in the absence of light. The white precipitate obtained was filtered off and washed with water (15 mL) and diethyl ether (10 mL). Yield: 0.08 g (80%). [Rh_2_(μ-O_2_CCMe_3_)_4_(HO_2_CCMe_3_)_2_] was prepared following a published procedure [40,41]. The rest of the reagents were purchased from commercial sources and used as received without further purification.

### 2.2. Physical Measurements

Elemental analyses were carried out by the Microanalytical Services of the Universidad Complutense de Madrid. FTIR spectra were measured using a Perkin–Elmer Spectrum 100 with a universal ATR accessory in the 4000–650 cm^−1^ spectral range. Mass spectrometry measurements were carried out by the Mass Spectrometry Services of the Universidad Complutense de Madrid. Electrospray ionization (ESI) mass spectra were recorded using an ion trap-Bruker Esquire-LC spectrometer. Matrix Assisted Laser Desorption Ionization-Time of Flight (MALDI-TOF) were carried out using a Bruker ULTRAFLEX spectrometer. NMR spectra were recorded at the NMR service of the Universidad Complutense de Madrid using a Bruker DPX 300 MHz-BACS-60 and a Bruker AV 500 MHz instrument.

### 2.3. Crystallography

Single crystal X-ray diffraction measurements were carried out at room temperature using a Bruker Smart-CCD diffractometer with a Mo Kα (λ = 0.71073 Å) radiation and a graphite monochromator. CCDC 1884924–1884932 contain the crystallographic data for the compounds described in this work. These data can be obtained free of charge from the Cambridge Crystallographic Data Centre via www.ccdc.cam.ac.uk/data_request/cif. A summary of some crystal and refinement data are shown in Table 1 and Table 2.

### 2.4. Synthesis

#### 2.4.1. Synthesis of [Rh_2_(μ-O_2_CPh)_4_(OEt_2_)_2_] (**1a**) and [Rh_2_(μ-O_2_CPh)_4_(OCMe_2_)_2_] (**1b**)

Synthesis of [Rh_2_(μ-O_2_CPh)_4_(OEt_2_)_2_] (**1a**): A mixture of 0.90 mmol (0.40 g) of [Rh_2_(μ-O_2_CMe)_4_] and benzoic acid (5 g) was refluxed for 30 min under nitrogen atmosphere. After cooling the reaction mixture, a dark green solid was obtained at the bottom of the flask while the walls of the flask were covered by sublimated acid. The acid was manually removed and the dark green solid was collected, milled and profusely washed with Et_2_O/CCl_4_ (1:1). Yield: 0.42 g (56%). Anal. Calcd. (%) for **1a**–1Et_2_O: C, 50.28; H, 3.96. Found (%): C, 50.02; H, 4.57. FT-IR (cm^−1^): 3069w, 2973w, 2878w, 1601s, 1563s, 1495w, 1443w, 1392s, 1159m, 1060m, 1028m, 934w, 912w, 845m, 831w, 811w, 787w, 716s, 691s. ESI^+^, *m/z* = 713, [Rh_2_(μ-O_2_CPh)_4_] + Na^+^. ^1^H-NMR: (500 MHz, acetone-d_6_, δ (ppm)): 7.57 (m, 20H), 3.40 (q, 8H), 1.11 (t, 12H).

Synthesis of [Rh_2_(μ-O_2_CPh)_4_(OCMe_2_)_2_] (**1b**): A few crystals suitable for single crystal X-ray diffraction were obtained by slow evaporation of a solution of **1a** in acetone.

#### 2.4.2. Synthesis of [Rh_2_(μ-O_2_CCH_2_OEt)_4_(HO_2_CCH_2_OEt)_2_] (**2a**) and [Rh_2_(μ-O_2_CCH_2_OEt)_4_(THF)_2_] (**2b** and **2b′**)

Synthesis of [Rh_2_(μ-O_2_CCH_2_OEt)_4_(HO_2_CCH_2_OEt)_2_] (**2a**): 0.68 mmol (0.30 g) of [Rh_2_(μ-O_2_CMe)_4_] in 2.8 mL of ethoxyacetic acid were stirred and heated at 150 °C for three hours under nitrogen atmosphere. The green solution obtained was concentrated to one third of its volume and 6 mL of diethyl ether and 30 mL of petroleum ether were added. Single crystals suitable for X-ray diffraction were obtained after keeping the solution four days in the fridge. Yield: 0.34 g (60%). FT-IR (cm^−1^): 3450w, 2979w, 2874w, 1672w, 1600s, 1434m, 1410s, 1367m, 1326s, 1265m, 1235m, 1116s, 1030m, 1009m, 986w, 894w, 840m, 732s.

Synthesis of [Rh_2_(μ-O_2_CCH_2_OEt)_4_(THF)_2_] (**2b** and **2b′**): 0.39 mmol (0.32 g) of compound **2a** were dissolved in 4 mL of THF and 30 mL of petroleum ether. The resultant solution was kept in fridge for two days. Bluish green single crystals suitable for X-ray diffraction were obtained. Yield: 0.16 g (54%). Anal. Calcd (%) for **2b**−2THF: C, 31.09; H, 4.57. Found (%): C, 30.04; H, 4.27. FT-IR (cm^−1^): 3450w, 2978w, 2876w, 1600s, 1436m, 1410s, 1367m, 1327s, 1266m, 1119s, 1082s, 1028m, 1010m, 928w, 895w, 840m, 733s. ESI^−^, *m*/*z* = 618, [Rh_2_(μ-O_2_CCH_2_OEt)_4_]^−^ and ESI^+^, *m*/*z* = 1133, [Rh_4_(μ-O_2_CCH_2_OEt)_7_]^+^. ^1^H-NMR: (300 MHz, CDCl_3_, δ (ppm)): 3.96 (s, 8H), 3.44 (q, 8 H), 1.16 (t, 12 H). A careful observation of the crystals allows the distinction of two different crystallization habits: prisms (**2b**) and thin plates (**2b′**).

#### 2.4.3. Synthesis of (PPh_4_)_n_[Rh_2_(μ-O_2_CMe)_4_Ag(CN)_2_]_n_ (**3a**) and {(PPh_4_)[Rh_2_(μ-O_2_CMe)_4_Ag(CN)_2_]·2CH_2_Cl_2_}_n_ (**3b**)

Synthesis of (PPh_4_)_n_[Rh_2_(μ-O_2_CMe)_4_Ag(CN)_2_]_n_ (**3a**): A solution of 0.1 mmol (0.05 g) of PPh_4_[Ag(CN)_2_] in 8 mL of acetone was added to a solution of 0.1 mmol (0.044 g) of [Rh_2_(μ-O_2_CMe)_4_] in 10 mL of diethyl ether. The colour changes immediately from green to greyish blue. After 24 h of stirring in absence of light, the pale violet precipitate was separated by filtration. Yield: 0.06 g (64%). Slow evaporation of a MeOH/acetone solution (1:1) of **3a** at room temperature yielded single crystals suitable for X-ray diffraction. Anal. Calcd. (%) for **3a**: C, 43.38; H, 3.43; N, 2.98. Found (%): C, 42.50; H, 3.40; N, 3.04. FT-IR (cm^−1^): 3089w, 3064w, 3006w, 2972w, 2927w, 2157w, 1598s, 1484m, 1426s, 1410s, 1343m, 1192w, 1165w, 1108s, 1042m, 1024m, 997m, 759m, 721s, 689s. MALDI^−^, *m*/*z* = 601, [Rh_2_(O_2_CMe)_4_Ag(CN)_2_]^−^. ^1^H-NMR: (500 MHz, DMSO-*d*_6_, δ (ppm)): 7.57 (m, 20H), 2.08 (s, 12H).

Synthesis of {(PPh_4_)[Rh_2_(μ-O_2_CMe)_4_Ag(CN)_2_]·2CH_2_Cl_2_}_n_ (**3b**): Slow diffusion of a 12 mL THF solution of [Rh_2_(μ-O_2_CMe)_4_] (0.16 mmol, 0.07 g) in a 8 mL solution of PPh_4_[Ag(CN)_2_] (0.16 mmol, 0.08 g) in dichloromethane yielded a few crystals suitable for X-ray diffraction after 1 day. Anal. Calcd. (%) for **3b**–1CH_2_Cl_2_: C, 40.96; H, 3.34; N, 2.73. Found (%): C, 40.67; H, 3.51; N, 2.73.

#### 2.4.4. Synthesis of (PPh_4_)_n_[Rh_2_(μ-O_2_CPh)_4_Ag(CN)_2_]_n_ (**4**)

Compound **4** was obtained following a similar procedure to that employed to obtain **3a** but using 0.1 mmol (0.084 g) of **1a** instead of [Rh_2_(μ-O_2_CMe)_4_]. Yield: 0.11 g (92%). Slow evaporation of a Et_2_O/acetone solution (1:1) at room temperature of **4** yielded single crystals suitable for X-ray diffraction. Anal. Calcd. (%) for **4**: C, 54.52; H, 3.39; N, 2.35. Found (%): C, 53.83; H, 3.43; N, 2.43. FT-IR (cm^−1^): 3062w, 2137w, 1603s, 1563s, 1493w, 1485w, 1438m, 1391s, 1173m, 1108s, 1070m, 1026m, 997m, 845m, 755m, 715s, 688s. ESI^−^, *m*/*z* = 716, [Rh_2_(μ-O_2_CPh)_4_CN]^−^; 851, [Rh_2_(μ-O_2_CPh)_4_Ag(CN)_2_]^−^; 1406, [Rh_4_(μ-O_2_CPh)_8_(CN)]^−^; ^1^H-NMR: (500 MHz, DMSO-*d*_6_, δ (ppm)): 7.65 (m, 40H).

#### 2.4.5. Synthesis of (PPh_4_)_n_[Rh_2_(μ-O_2_CCH_2_OEt)_4_Ag(CN)_2_]_n_ (**5**)

0.1 mmol (0.076 g) of **2b** and 0.10 mmol (0.052 g) of PPh_4_[Ag(CN)_2_] were dissolved separately in 8 and 12 mL of THF, respectively. Both solutions were mixed and stirred at room temperature for 24 h giving a violet precipitate, which was filtered of and washed with THF. Yield: 0.07 g (63%). Single crystals of **5** suitable for X-ray diffraction were obtained by slow diffusion of diethyl ether over a solution of the compound in dichloromethane. Anal. Calcd. (%) for **5**: C, 45.14; H, 4.33; N, 2.51. Found (%): C, 44.97; H, 4.21; N, 2.31. FT-IR (cm^−1^): 3075w, 2927m, 2972m, 2880m, 2154w, 1613s, 1485m, 1433s, 1408s, 1363m, 1320s, 1261m, 1163m, 1134s, 1107s, 1032m, 998m, 894w, 851s, 757m, 724s, 694s. ESI^−^, *m*/*z* = 619, [Rh_2_(μ-O_2_CCH_2_OEt)_4_]^−^ and ESI^+^, *m*/*z* = 339, [PPh_4_]^+^. ^1^H-NMR: (300 MHz, CDCl_3_, δ (ppm)): 7.81 (m, 20H), 3.85 (s, 8H), 3.31 (q, 8H), 1.05 (t, 12H).

#### 2.4.6. Synthesis of (PPh_4_)_2_{Rh_2_(μ-O_2_CCMe_3_)_4_[Ag(CN)_2_]_2_} (**6**)

The synthetic procedure to obtain **6** is analogous to that of **3** using [Rh_2_(μ-O_2_CCMe_3_)_4_(HO_2_CCMe_3_)_2_] (0.1 mmol, 0.08 g) instead of [Rh_2_(μ-O_2_CMe)_4_]. Yield: 0.065 g (81%). Single crystals suitable for X-ray diffraction were obtained by slow evaporation at room temperature of a solution of **6** in an acetone/THF (1:1) mixture. Anal. Calcd. (%) for **6**: C, 53.75; H, 4.76; N, 3.48. Found (%): C, 52.86; H, 4.67; N, 3.41. FT-IR (cm^−1^): 2961w, 2928w, 2921w, 2866w, 2135w, 1584s, 1482m, 1437m, 1411s, 1360s, 1221s, 1163w, 1109s, 1029w, 997m, 893m, 801w, 781w, 765m, 755m, 721s, 688s. ESI^−^, *m*/*z* = 769, [Rh_2_(μ-O_2_CCMe_3_)_4_Ag(CN)_2_]^−^; 1246, [Rh_4_(μ-O_2_CCMe_3_)_8_CN]^−^. ^1^H-NMR: (500 MHz, DMSO-*d*_6_, δ (ppm)): 7,84 (m, 40H), 0.89 (s, 36H).

## 3. Results and Discussion

### 3.1. Synthesis and Spectroscopic Characterization

[Rh_2_(μ-O_2_CPh)_4_(OEt_2_)_2_)] (**1a**) was obtained by metathesis reaction of [Rh_2_(μ-O_2_CMe)_4_] using melted benzoic acid as solvent. The recrystallization of **1a** in acetone led to crystals of [Rh_2_(μ-O_2_CPh)_4_(OCMe_2_)_2_] (**1b**). A similar reaction using ethoxyacetic acid led to [Rh_2_(μ-O_2_CCH_2_OEt)_4_(HO_2_CCH_2_OEt)_2_] *(***2a**). This compound was dissolved in THF and the solution was layered with petroleum ether. Crystals of [Rh_2_(μ-O_2_CCH_2_OEt)_4_(THF)_2_] (**2b** and **2b′**) were obtained from this solution.

The syntheses of the one-dimensional coordination polymers, (PPh_4_)_n_[Rh_2_(μ-O_2_CR)_4_Ag(CN)_2_]_n_ (R = Me (**3a**), Ph (**4**), CH_2_OEt (**5**)) and the non-polymeric compound, (PPh_4_)_2_{[Rh_2_(μ-O_2_CCMe_3_)_4_[Ag(CN)_2_]_2_} (**6**) were carried out by stirring for 24 h a solution of the corresponding starting [Rh_2_(μ-O_2_CR)_4_L_2_] compound with 1 equivalent of PPh_4_[Ag(CN)_2_] at room temperature. An appropriate solvent or solvent mixture was employed depending on the solubility of the starting dirhodium complex. Thus, a diethyl ether solution of the dirhodium compound and an acetone solution of the silver complex were used in the synthesis of **3a**, **4** and **6** while a THF solution of both reactants was used for **5**. Layering a THF solution of [Rh_2_(μ-O_2_CMe)_4_] on top of a dichloromethane solution of PPh_4_[Ag(CN)_2_] led to the formation of {(PPh_4_)[Rh_2_(μ-O_2_CMe)_4_Ag(CN)_2_]·2CH_2_Cl_2_}_n_ (**3b**). The same synthetic conditions employed to prepare **3a** and **4** gave rise to **6**, whose structure is not polymeric (see below).

Good elemental analyses were obtained for most of the complexes. However, in the case of compound **2a** no satisfactory elemental analyses have been obtained and for compounds **2b** and **3a** a difference >0.5 between the calculated and experimental values is observed. The quick loss of the solvent molecules not strongly bonded to the axial positions or the presence of a small impurity of [Rh_2_(μ-O_2_CR)_4_(solvent)_2_] in **2a** and **3a** could explain these discrepancies. This hypothesis is compatible with the NMR spectra of these compounds that do not show additional bands due to impurities.

The IR spectra of **1**–**6** display bands corresponding to the symmetric and antisymmetric stretching modes of the carboxylate groups: ν(COO)_a_ (1613–1584 cm^−1^) and ν(COO)_s_ (1433–1391 cm^−1^). The C-H stretching bands of the aliphatic groups appear below 3000 cm^−1^ while those of the aromatic groups of the carboxylate ligands or the (PPh_4_)^+^ cations appear above 3000 cm^−1^. Moreover, a ν(C≡N) band is observed in the 2135–2157 cm^−1^ range in the spectra of **3**–**6**.

The ^1^H-NMR spectrum of **1a** measured in acetone-d_6_ shows a multiplet structure at 7.57 ppm because of the 20 aromatic protons of its four PhCO_2_^−^ ligands. The spectrum also shows a triplet and a quartet signal at 1.11 and 3.40 ppm, respectively, corresponding to the signals of two Et_2_O molecules coordinated to the axial positions of the dirhodium units. The ^1^H-NMR spectrum of **4** measured in DMSO-*d*_6_ only displays a multiplet signal centred at 7.65 ppm corresponding to the 40 aromatic protons of the PhCO_2_^−^ ligands and (PPh_4_)^+^ cations.

The ^1^H-NMR spectra of **2b** and **5** measured in CDCl_3_ display a singlet (3.96 ppm for **2b** and 3.85 ppm for **5**), a quartet (3.44 for **2b** and 3.31 ppm for **5**) and a triplet (1.16 for **2b** and 1.05 ppm for **5**) due to the protons of the four EtOCH_2_CO_2_^−^ ligands. The spectrum of **5** also displays a multiplet signal centred a 7.81 ppm corresponding to the aromatic signals of the (PPh_4_)^+^ cations.

The ^1^H-NMR spectra of **3** and **6** measured in DMSO-*d*_6_ display a singlet at 2.08 ppm and 0.89 ppm, respectively. These signals correspond to the protons of the methyl groups of the MeCO_2_^−^ ligands of **3** and the Me_3_CCO_2_^−^ ligands of **6**. Both spectra also show a multiplet signal that corresponds to the aromatic protons of the (PPh_4_)^+^ cations. This multiplet is observed at 7.57 and 7.84 ppm for **3a** and **6**, respectively. The integration of these signals is consistent with the presence of one and two (PPh_4_)^+^ cations per formula for **3** and **6**, respectively.

### 3.2. Crystal Structures

The crystal structures of complexes **1b**, **2a**, **2b, 2b′**, **3a**, **3b**, **4**, **5** and **6** have been determined using the data obtained by single crystal X-ray diffraction measurements. The structure of all the compounds contains two rhodium atoms bridged by four carboxylate ligands with a typical paddlewheel arrangement. The axial positions are occupied by solvent molecules in the case of **1**–**2** and [Ag(CN)_2_]^−^ ions in the case of **3**–**6**. The metal-metal distances are in the 2.381–2.413 Å range in complexes **1**–**6** and are typical values of a single Rh-Rh bond (2.35–2.45 Å) [2].

The analysis of the Rh-O distances in **1b**, **2a**, **2b** and **2b′**, shows that the Rh-O_equatorial_ bond (average distance of 2.038 Å) is considerably stronger than the Rh-O_axial_ bond (average distance of 2.311 Å). Thus, these compounds are good starting materials to prepare coordination polymers because the solvent molecules placed in the axial positions can be easily replaced by bridging ligands connecting dirhodium units.

**Figure 2:**
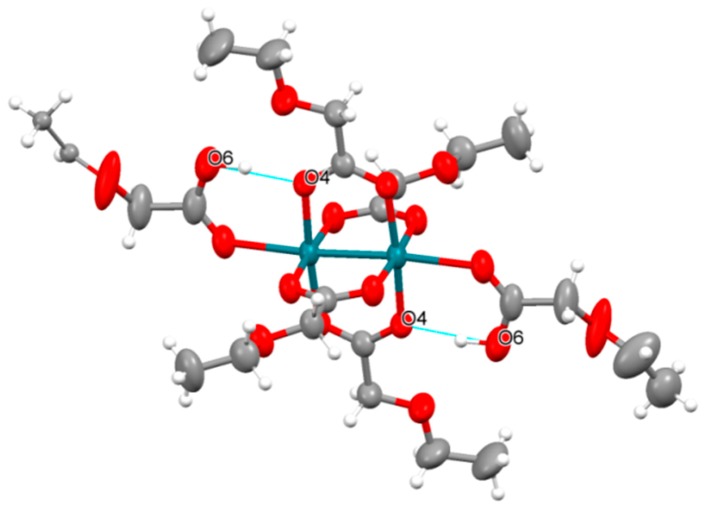
Representation of the paddlewheel unit of **2a** (50% probability ellipsoids) showing the intramolecular hydrogen bonds (blue line) present in this compound. Rhodium: green; oxygen: red; carbon: grey; hydrogen: white.

CH····π interactions and π-π stacking are observed in the structure of **1b** (See Figure S1). However, no strong intermolecular interactions are observed in the crystal structures of **2a**, **2b** and **2b′**. Additionally, intramolecular hydrogen bonds are observed between the hydrogen atom of the carboxylic acid of the EtOCH_2_CO_2_H ligands coordinated to the axial positions and the O4 of the EtOCH_2_CO_2_^−^ ligands in the equatorial positions in the structure of **2a** (Figure 2). The C-O distances in the carboxylic acid of the axial EtOCH_2_CO_2_H are 1.237 and 1.309 Å, corresponding to a double and single bond, respectively. This fact indicates that the axial ligands of **2a** are protonated and that the oxidation state of this paddlewheel unit is Rh_2_^4+^. The two axial EtOCH_2_CO_2_H molecules of **2a** are easily replaced by two THF molecules by dissolving the compound in a THF/petroleum ether mixture. Interestingly, this compound crystallized giving two different crystal structures (**2b** and **2b′**) (Table 1).

The crystal structure of **3a**, **3b**, **4** and **5** is formed by neutral dirhodium paddlewheel units that are coordinated to the nitrogen atom of [Ag(CN)_2_]^−^ bridging units forming anionic one-dimensional coordination polymers. The Rh-N distances are very similar in these complexes being in the 2.200–2.233 Å range. These distances are also similar to those observed in analogous gold derivatives [32].

**Figure 3:**
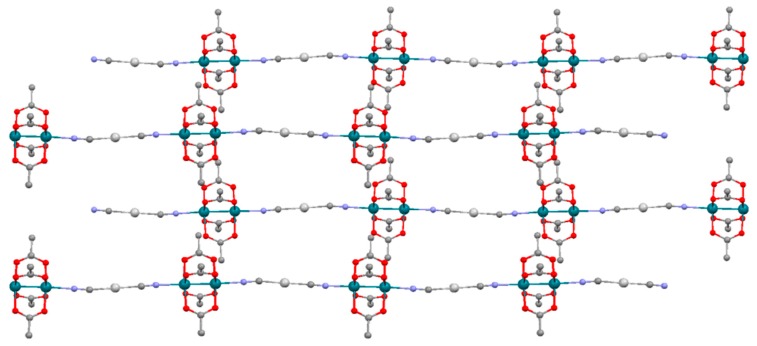
View of the chains that form **3a** along the *a* axis in a 2 × 2 × 2 cell packing. Rhodium: green; oxygen: red; carbon: dark grey; nitrogen: purple; silver: pale grey. Hydrogen atoms are omitted for clarity.

**Figure 4:**
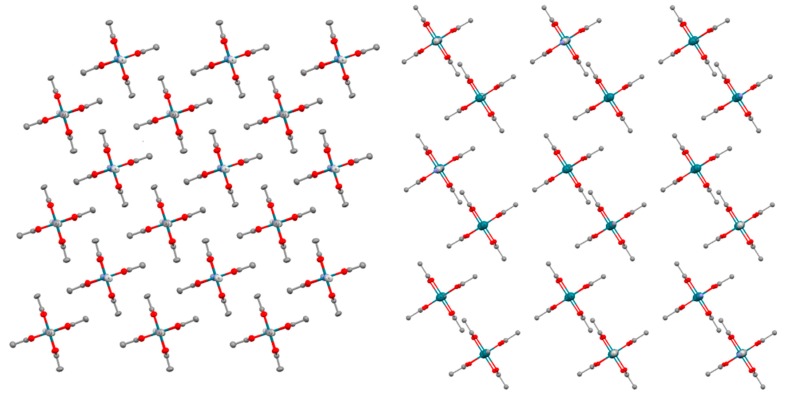
View of the chains that form **3a** along the *b* axis in a 3 × 3 × 3 cell packing (**left**). View of the chains that form **3b** along the *a* axis in a 3 × 3 × 3 cell packing (**right**). Hydrogen atoms are omitted for clarity.

The Rh-Ag-Rh angles in the chains of compounds **3a** and **3b** are very similar, being 176.08° and 178.25°, respectively. However, the values of Rh-N-C angles show a greater difference being 175.91 and 177.45° for **3a** and 171.33 and 170.60° for **3b** (Figure 3 and Figure S2). The average O-Rh-Rh-O torsion angles are 1.10° and 0.95°, respectively. The chains are placed parallel to each other. Each chain is surrounded by four other chains in the structure of **3a** while the structure of **3b** is formed by pairs of chains being surrounded by four pairs of chains (Figure 4). The (PPh_4_)^+^ cations in the structure of **3a** and the (PPh_4_)^+^ cations and dichloromethane molecules in the structure of **3b** are placed between chains (Figures S3 and S4). The packing does not allow the existence of Ag-Ag interactions between chains. Thus, the closest interchain Ag-Ag distances are 8.681 and 8.169 Å in **3a** and 8.976 Å in **3b**.

**Figure 5:**
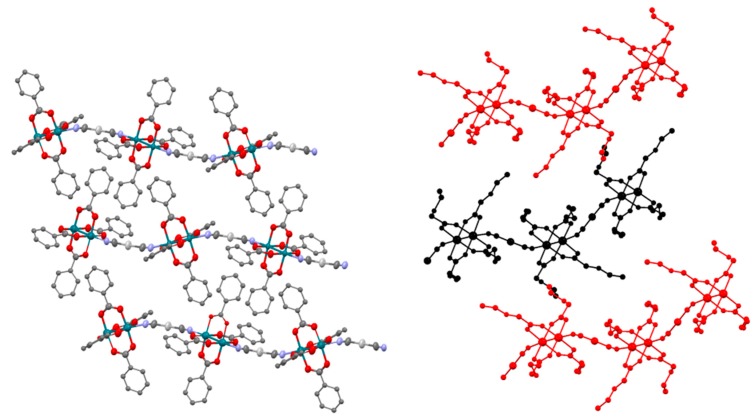
View of the chains that form **4** along the *b* axis (**left**). View of the chains that form **5** along the *a* axis. The chains are depicted in red and black colours for clarity (**right**). Hydrogen atoms are omitted for clarity.

Compounds **4** and **5** are also built by anionic chains that are formed by [Rh_2_(μ-O_2_CPh)_4_Ag(CN)_2_]^−^ and [Rh_2_(μ-O_2_CCH_2_OEt)_4_Ag(CN)_2_]^−^ monomers, respectively (Figure 5). The average O-Rh-Rh-O torsion angles are 1.00° and 2.17°, respectively. The Rh-Ag-Rh angle is 166.95° in complex **4** and 176.67° in complex **5** while the Rh-N-C angles are 165.29 and 172.97° in the case of **4** and 163.96 and 165.46° in the case of **5**. As a result of these parameters, the wavy structure of these two polymers is more pronounced than that of **3a** and **3b**. The chains are packed in a parallel disposition in both cases (Figures S5 and S6) with the (PPh_4_)^+^ cations placed between chains (Figures S7 and S8) and the shortest intermolecular Ag-Ag distances are 10.687 Å in **4** and 8.459 Å in **5**.

The analysis of the distances between the (PPh_4_)^+^ cations in the structures of **3a**, **3b**, **4** and **5** shows that there is no concerted multiple phenyl embraces similar to those described by Dance and Scudder [33]. Nevertheless, in **3a** the shortest P···P distance is 8.284 Å and there are crossed C-H····π interactions (3.520 and 3.740 Å) between phenyl rings of the pair of (PPh_4_)^+^ cations (Figure S9, top). These pairs are situated at 8.788 Å giving pseudo-chains (Figure S9, bottom). Similar interactions have been observed in **3b** and **5** (Figures S10 and S12) with P···P distances of 8.232 and 8.388 Å (for **3b**) and 8.562 and 8.734 Å (for **5**). The distances of the possible C-H····π interactions are at 3.194 Å for **3b** and 3.160 Å for **5**. Interestingly, the closest (PPh_4_)^+^ cations in **4** are at 10.387 Å with no significant interactions between them (Figure S11) but C-H····π interactions between (PPh_4_)^+^ cations and [Rh_2_(μ-O_2_CPh)_4_Ag(CN)_2_]^−^ units have been found (Figure S14).

Significant cation-anion interactions have been found only in compound **4**. In the other compounds, the interactions between the pair of cations are very important to determine their crystal structure.

**Figure 6:**
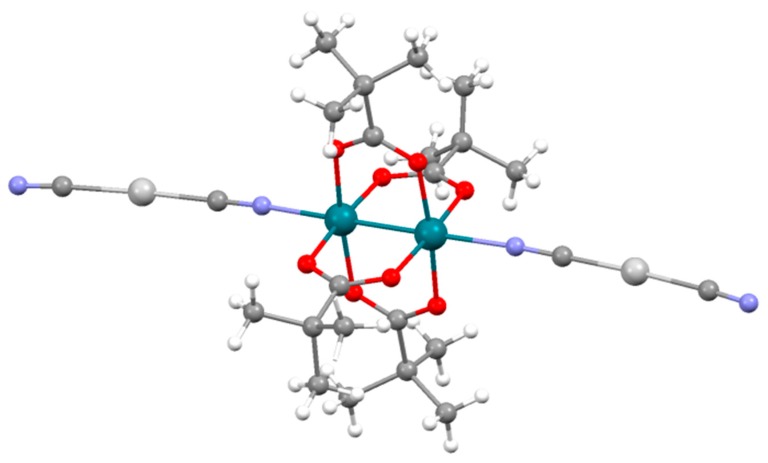
View of the {Rh_2_(μ-O_2_CCMe_3_)_4_[Ag(CN)_2_]_2_}^2−^ units that form **6**.

**Figure 7:**
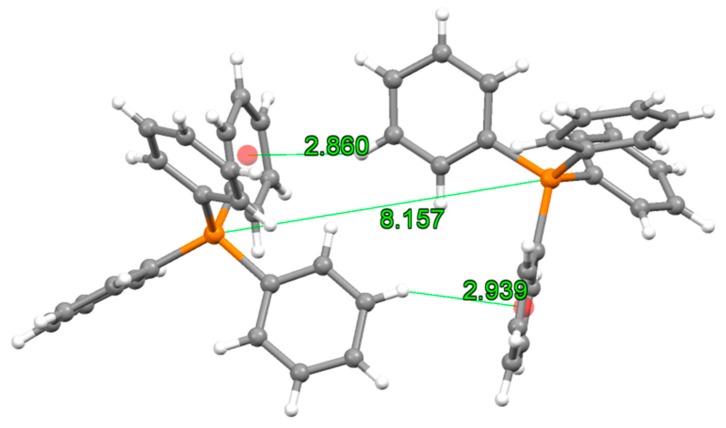
Interactions found between the closest (PPh_4_)^+^ cations in the structure of **6**. Distances are shown in Å.

The structure of **6** is formed by discrete {Rh_2_(μ-O_2_CCMe_3_)_4_[Ag(CN)_2_]_2_}^2−^ (Figure 6) and (PPh_4_)^+^ units. This compound was prepared following the same synthetic conditions employed to prepare **3a** and **4** whose structure is polymeric. One would expect that the formation of a coordination polymer should be more thermodynamically favourable. However, a polymer could not be obtained in this case despite of the efforts made. The formation of a molecular compound instead of a coordination polymer should be related with a more efficient packing than that of a potential coordination polymer made of the same building blocks. The analysis of the crystal structure of **6** did not reveal any strong dirhodium-(PPh_4_)^+^ interaction but C-H····π interactions are observed involving the closest (PPh_4_)^+^ cations in the structure. Particularly, this pair of cations displays two nearly vertex-to-face intermolecular interactions at 2.860 and 2.939 Å (Figure 7 and Figure S13). Moreover, we hypothesize that the higher solubility of the trimethylacetato derivative in acetone causes the complex to remain longer in the solution during the crystallization favouring the formation of **6** instead of a coordination polymer. A similar reasoning has been used to explain the molecular nature of [Ru_2_Cl(µ-O_2_CCMe_3_)_4_(OH_2_)] [42] and the polymeric structure found in [Ru_2_Cl(μ-O_2_CPh)_4_] and [Ru_2_Cl(μ-O_2_CMe)_4_] [43,44]. Interestingly, the ethoxyacetate ligand gives the polymeric **5** whereas in the diruthenium complexes, this ligand leads to [Ru_2_Cl(μ-O_2_CCH_2_OEt)_4_], which contains both polymeric and molecular fragments [45]. These data suggest a strong influence of the solvent and the nature of the equatorial ligand in the molecular or polymeric nature on the paddlewheel dinuclear complexes.

Taking into account the arrangement of these one-dimensional compounds in the solid state these complexes could exhibit functional properties such as gas occlusion or catalysis, similar to other three-dimensional compounds [46,47].

## 4. Conclusions

Anionic one-dimensional coordination polymers of the type (PPh_4_)_n_[Rh_2_(μ-O_2_CR)_4_Ag(CN)_2_]_n_ (R = Me (**3**), Ph (**4**), CH_2_OEt (**5**)) can be obtained using neutral dirhodium compounds and PPh_4_[Ag(CN)_2_]. The branched trimethylacetate ligand, which favours the solubility of the starting dirhodium complex, leads to the discrete heteronuclear complex (PPh_4_)_2_{Rh_2_(μ-O_2_CCMe_3_)_4_[Ag(CN)_2_]_2_} (**6**). The presence of bulky (PPh_4_)^+^ cations prevents the existence of intermolecular Ag-Ag interactions. The existence of C-H····π interactions between pairs of (PPh_4_)^+^ cations in **3a**, **3b**, **5** and **6** and significant cation-anion interactions in **4** have an important influence on the packing in solid state of these complexes.

## Figures and Tables

**Table 1:** Crystal and refinement data for **1**–**2** and **3a**.

	1b	2a	2b	2b′	3a
Formula	C_34_H_32_ O_10_Rh_2_	C_24_H_42_ O_18_Rh_2_	C_24_H_44_O_14_Rh_2_		C_34_H_32_AgN_2_ O_8_PRh_2_
fw	806.41	824.40	762.41		941.28
Space group	*C*2/c	*P*2_1_/c	*P*-1	*P*2_1_/c	*P*2_1_/n
*a*/Å	18.873(3)	8.1426(19)	8.687(5)	13.5323(13)	11.7168(8)
*b*/Å	15.669(3)	24.685(6)	13.558(5)	15.0682(15)	26.3945(17)
*c*/Å	23.988(4)	8.4475(19)	14.312(5)	8.0473(8)	11.7290(8)
*α*/°			71.929(5)		
*β*/°	103.999(15)	99.906(4)	84.838(5)	98.481(2)	93.595(1)
*α*/°			86.272(5)		
*V*/Å^3^	6883(2)	1672.7(7)	1594.8(12)	1623.0(3)	3620.2(4)
*Z*	8	2	2	2	4
*d* calc/g·cm^−3^	1.556	1.637	1.588	1.560	1.727
*μ*/mm^−1^	1.013	1.060	1.096	1.077	1.531
*R* indices	*R*_1_ = 0.0491 *wR*_2_ = 0.1590	*R*_1_ = 0.0402 *wR*_2_ = 0.1002	*R*_1_ = 0.0393 *wR*_2_ = 0.1093	*R*_1_ = 0.0335 *wR*_2_ = 0.0900	*R*_1_ = 0.0494 *wR*_2_ = 0.1687
GooF on *F*^2^	1.031	0.997	0.998	0.997	0.999

**Table 2:** Crystal and refinement data for **3b** and **4**–**6.**

	3b	4	5	6
Formula	C_36_H_36_AgCl_4_N_2_O_8_PRh_2_	C_54_H_40_Ag N_2_O_8_PRh_2_	C_42_H_48_Ag N_2_O_12_PRh_2_	C_72_H_75_Ag_2_ N_4_O_8_P_2_Rh_2_
fw	1111.13	1189.54	1117.48	1607.86
Space group	*P*-1	*P*-1	*P*2_1_/c	*Pnma*
*a*/Å	13.1180(12)	12.2802(9)	13.1982(13)	22.016(4)
*b*/Å	13.6197(13)	13.3534(9)	22.201(2)	33.450(2)
*c*/Å	13.6949(13)	17.3652(12)	16.9090(17)	11.691(6)
*α*/°	87.033(2)	83.105(1)		
*β*/°	78.278(2)	83.373(1)	111.788(2)	
*α*/°	79.928(2)	64.333(1)		
*V*/Å^3^	2358.5(4)	2541.6(3)	4600.6(8)	8610(5)
*Z*	2	2	4	4
*d* calc/g·cm^−3^	1.565	1.554	1.613	1.240
*μ*/mm^−1^	1.407	1.109	1.225	0.907
*R* indices	*R*_1_ = 0.0396 *wR*_2_ = 0.1187	*R*_1_ = 0.0587 *wR*_2_ = 0.1881	*R*_1_ = 0.0379 *wR*_2_ = 0.1040	*R*_1_ = 0.0686 *wR*_2_ = 0.1648
GooF on *F*^2^	0.995	1.008	1.042	0.993

## Supplementary Materials

The following are available online at http://www.mdpi.com/2073-4360/11/1/111/s1: Figure S1: Intermolecular interactions found in the structure of **1b**. Figure S2: View of the chains that form **3b** along the *c* axis in a 2 × 2 × 2 cell packing. Figure S3: Representation of the structure of **3a** along the *a* axis in a 2 × 2 × 2 cell packing. Figure S4: Representation of the structure of **3b** along the *c* axis in a 2 × 2 × 2 cell packing. Figure S5: View of the chains that form **4** along the direction of the Rh-Rh bond. Figure S6: View of the chains that form **5** along the direction of the Rh-Rh bond. Figure S7: Representation of the structure of **4** along the *a* axis: Figure S8: Representation of the structure of **5** along the *a* axis. Figure S9: Interactions found between the closest (PPh_4_)^+^ cations in the structure of **3a**. Figure S10: Interactions found between the closest (PPh_4_)^+^ cations in the structure of **3b**. Figure S11: Closest (PPh_4_)^+^ cations in the structure of **4**. Figure S12: Interactions found between the closest (PPh_4_)^+^ cations in the structure of **5**. Figure S13: Representation of the structure of **6** along the *c* axis and view of the interactions between the closest (PPh_4_)^+^ cations. Figure S14: Interactions found between the closest cations and anionic chains in the structure of **4**.

## References

[1] Cotton F.A., Walton R.A. (1982). Multiple Bonds between Metal Atoms.

[2] Cotton F.A., Murillo C., Walton R.A. (2005). Multiple Bonds between Metal Atoms.

[3] Liddle S.T. (2015). Molecular Metal-Metal Bonds: Compounds, Synthesis, Properties.

[4] Liao K., Liu W., Niemeyer Z.L., Ren Z., Bacsa J., Musaev D.G., Sigman M.S., Davies H.M.L. (2018). Site-Selective Carbene-Induced C–H Functionalization Catalyzed by DirhodiumTetrakis(triarylcyclopropanecarboxylate) Complexes. ACS Catal..

[5] Adly F.G. (2017). On the Structure of Chiral Dirhodium(II) Carboxylate Catalysts: Stereoselectivity Relevance and Insights. Catalysts.

[6] Berry J.F. (2015). Metal–metal multiple bonded intermediates in catalysis. J. Chem. Sci..

[7] Hansen J., Davies H.M.L. (2008). High symmetry dirhodium(II) paddlewheel complexes as chiral catalysts. Coord. Chem. Rev..

[8] Ohata J., Ball T.Z. (2018). Rhodium at the chemistry–biology interface. Dalton Trans..

[9] Jalilehvand F., Enriquez Garcia A., Niksirat P. (2017). Reactions of Antitumor Active Dirhodium(II) Tetraacetate Rh_2_(CH_3_COO)_4_ with Cysteine and Its Derivatives. ACS Omega.

[10] Knoll J.D., Turro C. (2015). Control and utilization of ruthenium and rhodium metal complex excited states for photoactivated cancer therapy. Coord. Chem. Rev..

[11] Peña B., Barhoumi R., Burghardt R.C., Turro C., Dunbar K.R. (2014). Confocal Fluorescence Microscopy Studies of a Fluorophore-Labeled Dirhodium Compound: Visualizing Metal–Metal Bonded Molecules in Lung Cancer (A549) Cells. J. Am. Chem. Soc..

[12] Leung C.-H., Zhong H.J., Chan D.S.H., Ma D.L. (2013). Bioactive iridium and rhodium complexes as therapeutic agents. Coord. Chem. Rev..

[13] Sarkar M., Daw P., Ghatak T., Bera J.K. (2014). Amide-Functionalized Naphthyridines on a Rh^II^–Rh^II^ Platform: Effect of Steric Crowding, Hemilability, and Hydrogen-Bonding Interactions on the Structural Diversity and Catalytic Activity of Dirhodium(II) Complexes. Chem. Eur. J..

[14] Amo-Ochoa P., Jiménez-Aparicio R., Perles J., Torres M.R., Gennari M., Zamora F. (2013). Structural Diversity in Paddlewheel Dirhodium(II) Compounds through Ionic Interactions: Electronic and Redox Properties. Cryst. Growth Des..

[15] Cotton F.A., Dikarev E.V., Petrukhina M.A., Schmitz M., Stang P.J. (2002). Supramolecular Assemblies of Dimetal Complexes with Polydentate N-Donor Ligands: From a Discrete Pyramid to a 3D Channel Network. Inorg. Chem..

[16] Kataoka Y., Yano N., Shimodaira T., Yan Y.-N., Yamasaki M., Tanaka H., Omata K., Kawamoto T., Handa M. (2016). Paddlewheel-Type Dirhodium Tetrapivalate Based Coordination Polymer: Synthesis, Characterization, and Self-Assembly and Disassembly Transformation Properties. Eur. J. Inorg. Chem..

[17] Fritsch N., Wick C.R., Waidmann T., Dral P.O., Tucher J., Heinemann F.W., Shubina T.E., Clark T., Burzlaff N. (2014). Multiply Bonded Metal(II) Acetate (Rhodium, Ruthenium, and Molybdenum) Complexes with the *trans*-1,2-Bis(*N*-methylimidazol-2-yl)ethylene Ligand. Inorg. Chem..

[18] Dikarev E.V., Shpanchenko R.V., Andreini K.W., Block E., Jin J., Petrukhina M.A. (2004). Powder Diffraction Study of a Coordination Polymer Comprised of Rigid Building Blocks: [Rh_2_(O_2_CCH_3_)_4_‚µ^2^-Se_2_C_5_H_8_-Se,Se′]_∞_. Inorg. Chem..

[19] Kim Y., Kim S.-J., Lough A.J. (2001). New dirhodium(II,II) carboxylates with 2,6-bis(N-1,2,4-triazolyl)pyridinato ligand (btp). Polyhedron.

[20] Gonzalez-Belman O.F., Yazmín Varela Y., Flores-Álamo M., Wrobel K., Gutierrez-Granados S., Peralta-Hernández J.M., Jiménez-Halla J.O.C., Serrano O. (2017). Microwave-Assisted Synthesis and Characterization of [Rh_2_(OAc)_4_(L)_2_] Paddlewheel Complexes: A Joint Experimental and Computational Study. Int. J. Inorg. Chem..

[21] Ye Q.-S., Li X.-N., Jin Y., Yu J., Chang Q.-W., Jiang J., Yan C.-X., Li J., Liu W.-P. (2015). Synthesis, crystal structures and catalytic activity of tetrakis(acetato)dirhodium(II) complexes with axial picoline ligands. Inorg. Chim. Acta.

[22] Cmoch P., Głaszczka R., Jaźwiński J., Kamieńskia B., Senkara E. (2014). Adducts of nitrogenous ligands with rhodium(II) tetracarboxylates and tetraformamidinate: NMR spectroscopy and density functional theory calculations. Magn. Reson. Chem..

[23] Heyduk A.F., Krodel D.J., Meyer E.E., Nocera D.G. (2002). A Luminescent Heterometallic Dirhodium−Silver Chain. Inorg. Chem..

[24] Uemura K. (2017). One-dimensional complexes extended by unbridged metal–metal bonds based on a HOMO–LUMO interaction at the d*_z_*^2^ orbital between platinum and heterometal atoms. Dalton Trans..

[25] Dikarev E.V., Andreini K.W., Petrukhina M.A. (2004). On the Road to a Termolecular Complex with Acetone:  A Heterometallic Supramolecular Network {[Rh_2_(O_2_CCF_3_)_4_]·μ_2_-OCMe_2_·[Cu_4_(O_2_CCF_3_)_4_]}. Inorg. Chem..

[26] Uemura K., Ebihara M. (2011). One-Dimensionally Extended Paddlewheel Dirhodium Complexes from Metal–Metal Bonds with Diplatinum Complexes. Inorg. Chem..

[27] Uemura K., Ebihara M. (2013). Paramagnetic One-Dimensional Chains Comprised of Trinuclear Pt–Cu–Pt and Paddlewheel Dirhodium Complexes with Metal–Metal Bonds. Inorg. Chem..

[28] Uemura K., Kanbara T., Ebihara M. (2014). Two Types of Heterometallic One-Dimensional Alignment Composed of Acetamidate-Bridged Dirhodium and Pivalamidate-Bridged Diplatinum Complexes. Inorg. Chem..

[29] Uemura K., Yamada T., Kanbara T., Ebihara M. (2015). Acetamidate-bridged paddlewheel dirhodium complex sandwiched by mononuclear platinum complexes with axial metal–metal bonds affording neutral heterometallic one-dimensional alignments. Inorg. Chim. Acta.

[30] Yamada T., Ebihara M., Uemura K. (2016). Heterometallic one-dimensional chain with tetradeca metal repetition constructed by amidate bridged dirhodium and pivalate bridged diplatinum complexes influenced by hydrogen bonding. Dalton Trans..

[31] Uemura K. (2018). Magnetic behavior in heterometallic one-dimensional chains or octanuclear complex regularly aligned with metal-metal bonds as –Rh-Rh-Pt-Cu-Pt. J. Mol. Struct..

[32] Amo-Ochoa P., Delgado S., Gallego A., Gómez-García C.J., Jiménez-Aparicio R., Martínez G., Perles J., Rosario Torres M.R. (2012). Structure and Properties of One-Dimensional Heterobimetallic Polymers Containing Dicyanoaurate and Dirhodium(II) Fragments. Inorg. Chem..

[33] Dance I., Scudder M. (1996). Supramolecular Motifs: Concerted Multiple Phenyl Embraces between Ph_4_P^+^ Cations Are Attractive and Ubiquitous. Chem. Eur. J..

[34] Dance I., Scudder M. (1996). Concerted supromolecular motifs: Linear columns and zigzag chains of multiple phenyl embraces involving PPh_4_P^+^ cations in crystals. J. Chem. Soc. Dalton Trans..

[35] Scudder M., Dance I. (2000). Sixfold phenyl embraces with substituted phenyl in PPh_3_. J. Chem. Soc. Dalton Trans..

[36] Dance I., Scudder M. (1998). Crystal supramolecularity: Elaborate six-, eight- and twelve-fold concerted phenyl embraces in compounds [M(PPh_3_)_3_]^z^ and [M(PPh_3_)_4_]^z^. New J. Chem..

[37] Ali B., Dance I., Scudder M., Craig D. (2002). Dimorphs of (Ph_4_P)_2_[Cd_2_(SPh)_6_]: Crystal Packing Analyses and the Interplay of Intermolecular and Intramolecular Energies. Cryst. Growth Des..

[38] Janssen F.F.B.J., de Gelder R., Rowan A.E. (2011). 2. The Multiple Phenyl Embrace as a Synthon in Cu(I)/PPh_3_/N-Donor Ligand Coordination Polymers. Cryst. Growth Des..

[39] Zaręba J.K., Białek M.J., Janczak J., Zoń J., Dobosz A. (2014). Extending the Family of Tetrahedral Tectons: Phenyl Embraces in Supramolecular Polymers of Tetraphenylmethane-based Tetraphosphonic Acid Templated by Organic Bases. Cryst. Growth Des..

[40] Cotton F.A., Felthouse T.R. (1980). Structural studies of three tetrakis(carboxylato)dirhodium(II) adducts in which carboxylate groups and axial ligands are varied. Inorg. Chem..

[41] Mikuriya M., Yamamoto J., Ishida H., Yoshioka D., Handa M. (2011). Preparation and Crystal Structure of Tetrakis(μ-pivalato-O,O′)bis[(pivalic acid-O)rhodium(II)]. X-Ray Struct. Anal. Online.

[42] Barral M.C., Jiménez-Aparicio R., Priego J.L., Royer E.C., Saucedo M.J., Urbanos F.A., Amador U. (1995). Non-polymeric diruthenium(II,III) carboxylates. Crystal structures of [Ru_2_Cl(µ-O_2_CCMe_3_)_4_(H_2_O)] and [Ru_2_Cl(µ-O_2_CCHMe_2_)_4_(thf)] (thf = tetrahydrofuran). J. Chem. Soc. Dalton Trans..

[43] Masaaki A., Yoichi S., Tadashi Y., Tasuku I. (1992). X-Ray Structure, Ligand Substitution, and Other Properties of Trinuclear Ruthenium Complex, [Ru_3_(μ^3^-O)(μ-C_6_H_5_COO)_6_(py)_3_](PF_6_) (py = pyridine), and X-Ray Structure of Dinuclear Ruthenium Complex, [Ru_2_(μ-C_6_H_5_COO)_4_Cl]. Bull. Chem. Soc. Jpn..

[44] Bino A., Cotton F.A., Felthouse T.R. (1979). Structural studies of some multiply bonded dirutheniumtetracarboxylate compounds. Inorg. Chem..

[45] Barral M.C., Jiménez-Aparicio R., Priego J.L., Royer E.C., Urbanos F.A., Amador U. (1998). Diruthenium(II,III) Carboxylate Compounds: Existence of both Polymeric and Ionic Forms in Solution and Solid State. Inorg. Chem..

[46] Naito S., Tanibe T., Saito E., Miyao T., Moriet W. (2001). A Novel Reaction Pathway in Olefin-Deuterium Reaction inside the Microporous of Rh(II) Dicarboxylate Polymer Complexes. Chem. Lett..

[47] Nukada R., Mori W., Takamizawa S., Mikuriya M., Handa M., Naono H. (1999). Microporous Structure of a Chain Compound of Copper(II) Benzoate Bridged by Pyrazine. Chem. Lett..

