# Supplementary material for: Synthesis and Structural Characterization of a Series of One-Dimensional Heteronuclear Dirhodium-Silver Coordination Polymers
Source: Polymers (Basel). 2019 Jan 10;11(1):111. doi: 10.3390/polym11010111 (PMC6401823; doi:10.3390/polym11010111)
Supplement: Supplementary file 1 [file polymers-11-00111-s001.pdf]

Synthesis and Structural Characterization of a Series of One-dimensional Heteronuclear Dirhodium-Silver Coordination Polymers

Paula Cruz[‡,1], Estefania Fernandez-Bartolomé[‡,1], Miguel Cortijo*[1], Patricia Delgado-Martínez[2], Rodrigo González-Prieto[1], José L. Priego,*[1] M. Rosario Torres[2], and Reyes Jiménez-Aparicio*[1]

[1] Departamento de Química Inorgánica, Facultad de Ciencias Químicas, Universidad Complutense de Madrid, Ciudad Universitaria, E-28040 Madrid, Spain.

[2] Centro de Asistencia a la Investigación Difracción de Rayos X, Facultad de Ciencias Químicas, Universidad Complutense de Madrid, E-28040 Madrid, Spain.

* Correspondence: miguelcortijomontes@ucm.es (M.C); bermejo@quim.ucm.es (J.L.P.); reyesja@quim.ucm.es (R.J.-A.).

‡ Both authors contributed equally to this work.

TABLE OF CONTENTS

Figure S1. Intermolecular interactions found in the structure of **1b** 2

Figure S2. View of the chains that form **3b** along the *c* axis in a 2x2x2 cell packing 2

Figure S3. Representation of the structure of **3a** along the *a* axis in a 2x2x2 cell packing 3

Figure S4. Representation of the structure of **3b** along the *c* axis in a 2x2x2 cell packing 3

Figure S5. View of the chains that form **4** along the direction of the Rh-Rh bond 4

Figure S6. View of the chains that form **5** along the direction of the Rh-Rh bond 4

Figure S7. Representation of the structure of **4** along the *a* axis 5

Figure S8. Representation of the structure of **5** along the *a* axis 5

Figure S9. Interactions found between the closest $(PPh_4)^+$ cations in the structure of **3a** 6

Figure S10. Interactions found between the closest $(PPh_4)^+$ cations in the structure of **3b** 7

Figure S11. Closest $(PPh_4)^+$ cations in the structure of of **4** 8

Figure S12. Interactions found between the closest $(PPh_4)^+$ cations in the structure of **5** 9

Figure S13. Representation of the structure of **6** along the c axis and view of the interactions between the closest $(PPh_4)^+$ cations. 10

Figure S14. Interactions found between the closest cations and anionic chains in the structure of **4** 11

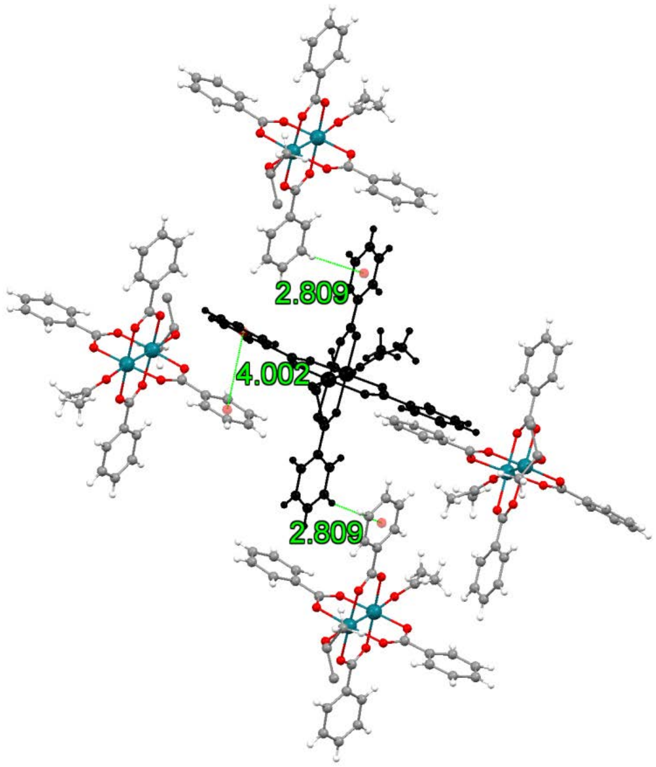

Figure S1. Intermolecular interactions found in the structure of **1b**. Thermal ellipsoids are omitted for clarity.

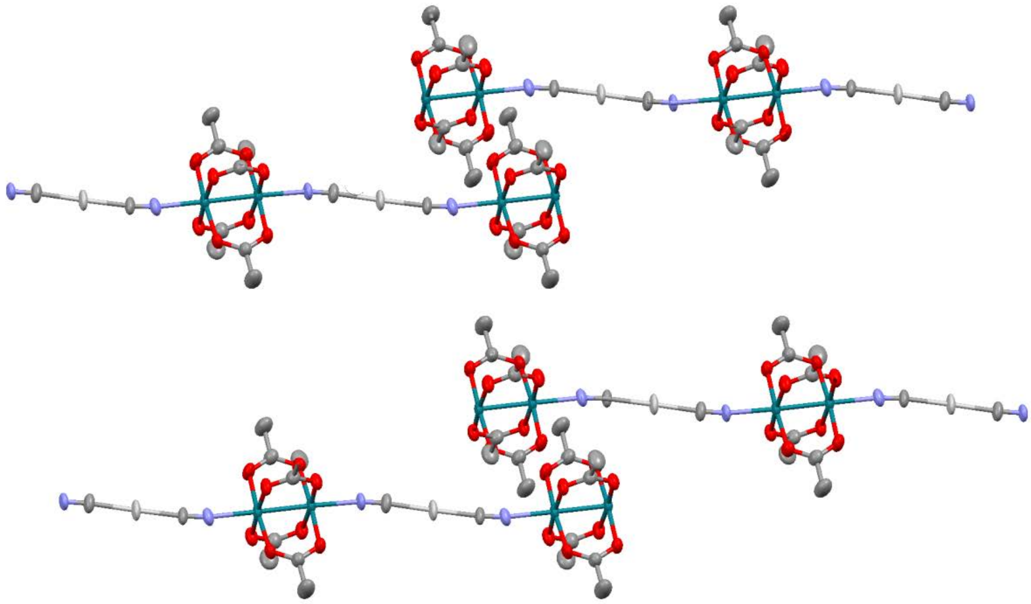

Figure S2. View of the chains that form **3b** along the *c* axis.

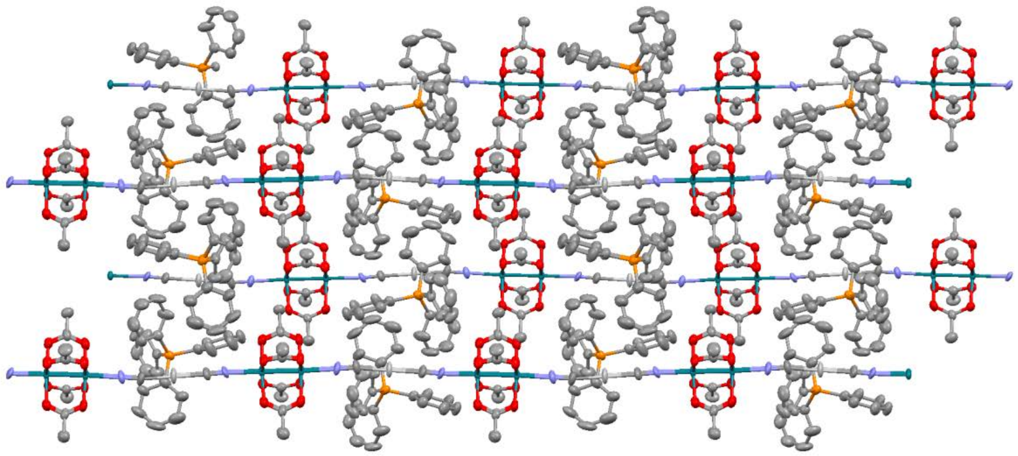

Figure S3. Representation of the structure of **3a** along the *a* axis.

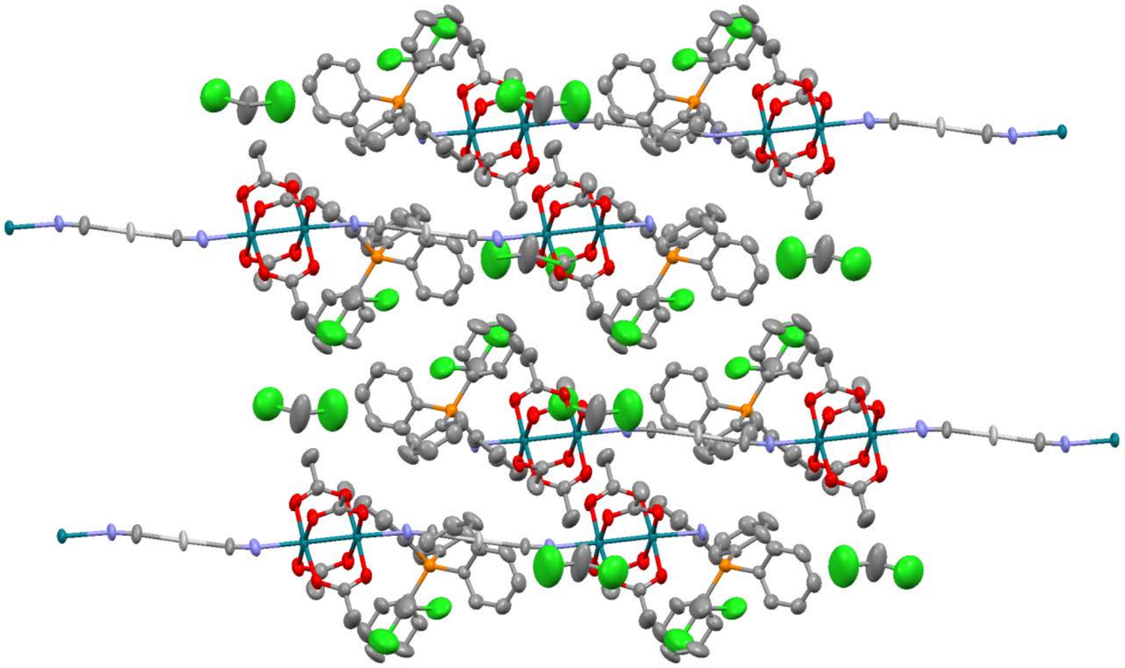

Figure S4. Representation of the structure of **3b** along the *c* axis.

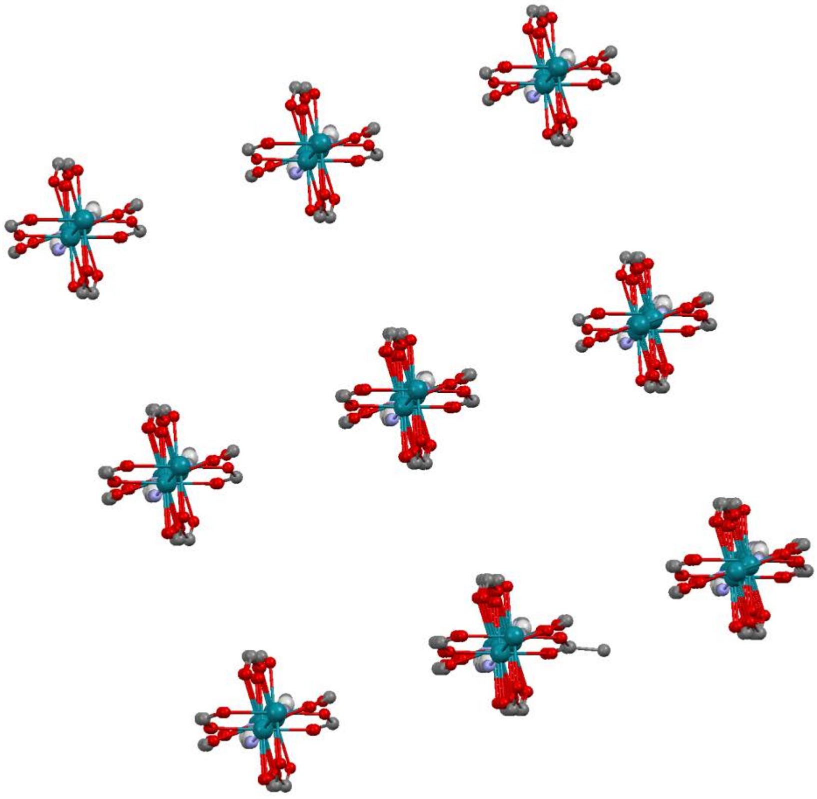

Figure S5. View of the chains that form **4** along the direction of the Rh-Rh bond. The substituent of the carboxylate groups have been omitted for clarity.

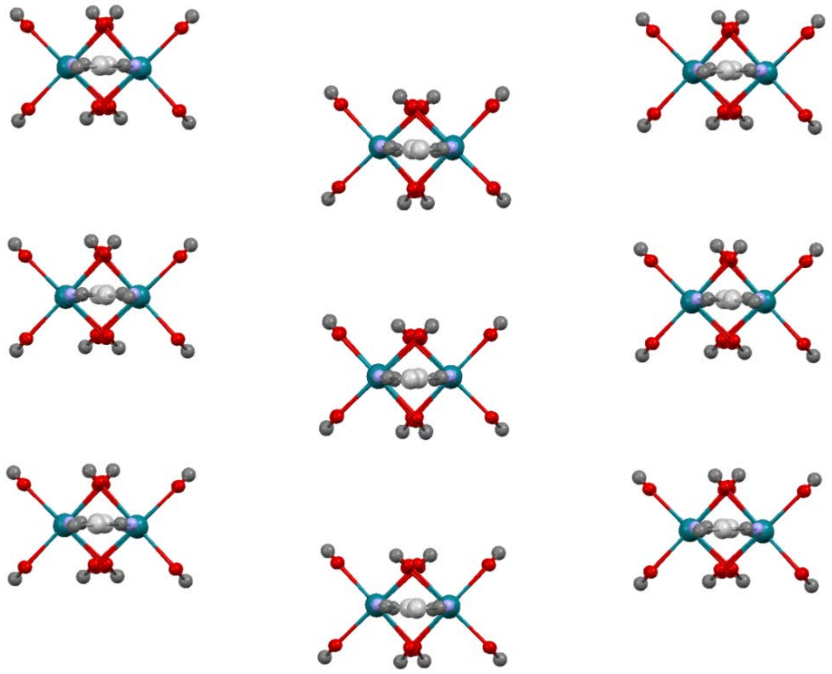

Figure S6. View of the chains that form **5** along the direction of the Rh-Rh bond. The substituent of the carboxylate groups have been omitted for clarity.

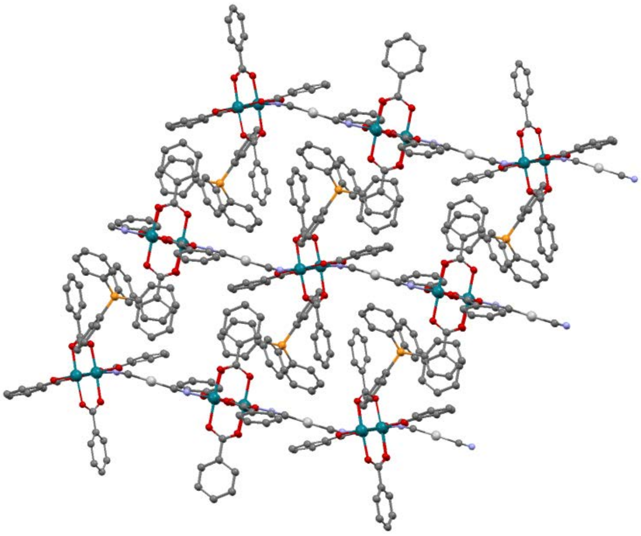

Figure S7. Representation of the structure of **4** along the *a* axis. Thermal ellipsoids are omitted for clarity.

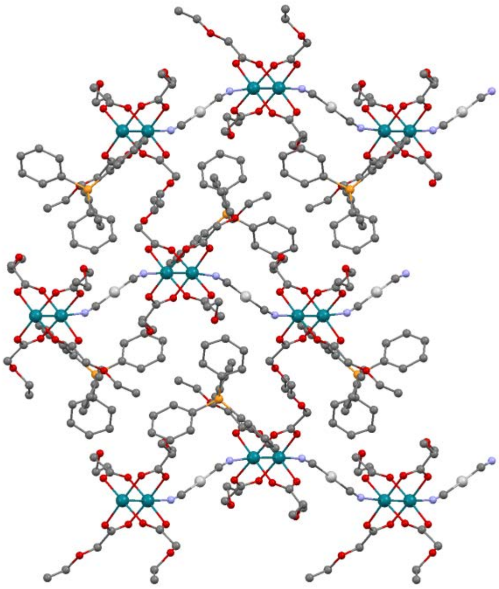

Figure S8. Representation of the structure of **5** along the *a* axis. Thermal ellipsoids are omitted for clarity.

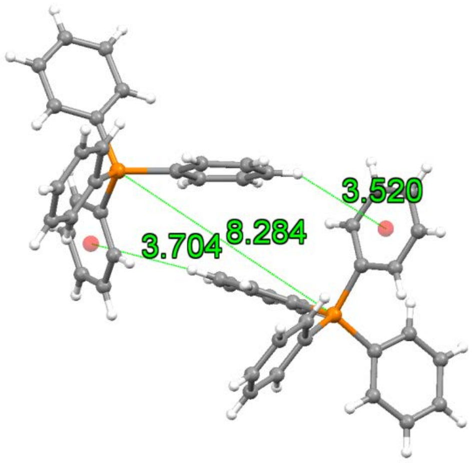

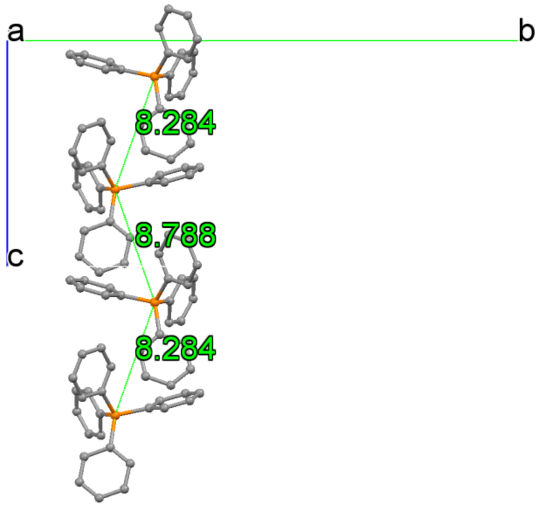

Figure S9. Interactions found between the closest $(PPh_4)^+$ cations in the structure of of **3a**.

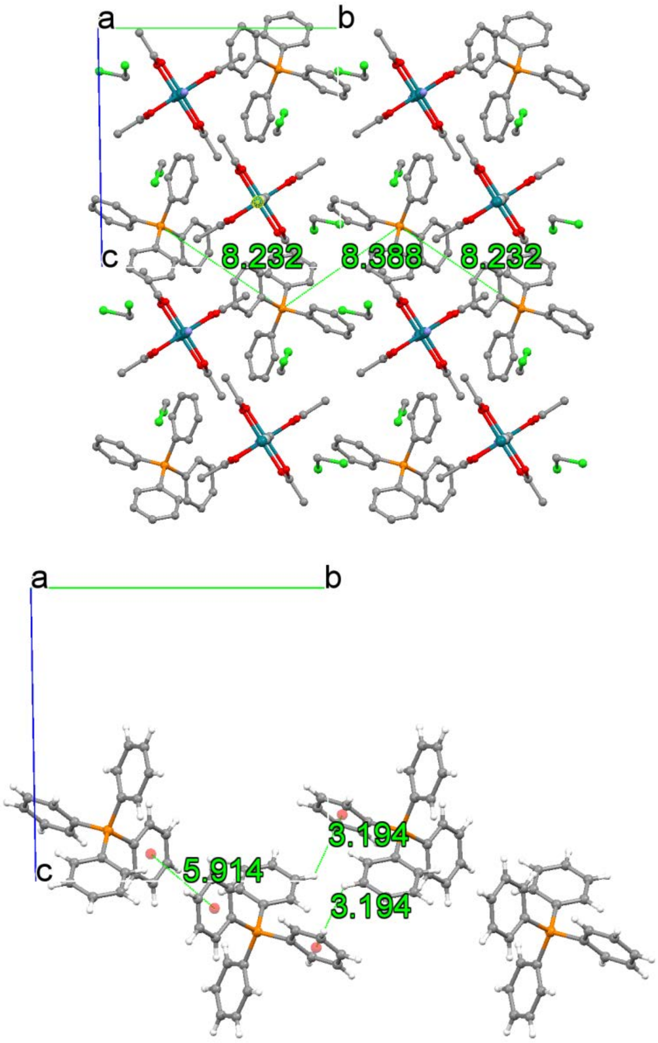

Figure S10. Interactions found between the closest $(PPh_4)^+$ cations in the structure of of **3b**.

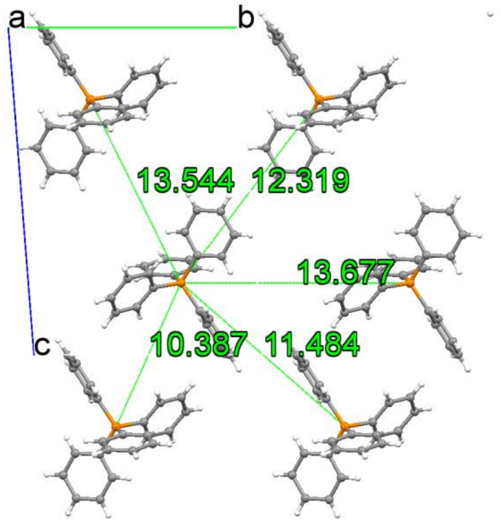

Figure S11. Closest $(PPh_4)^+$ cations in the structure of **4**

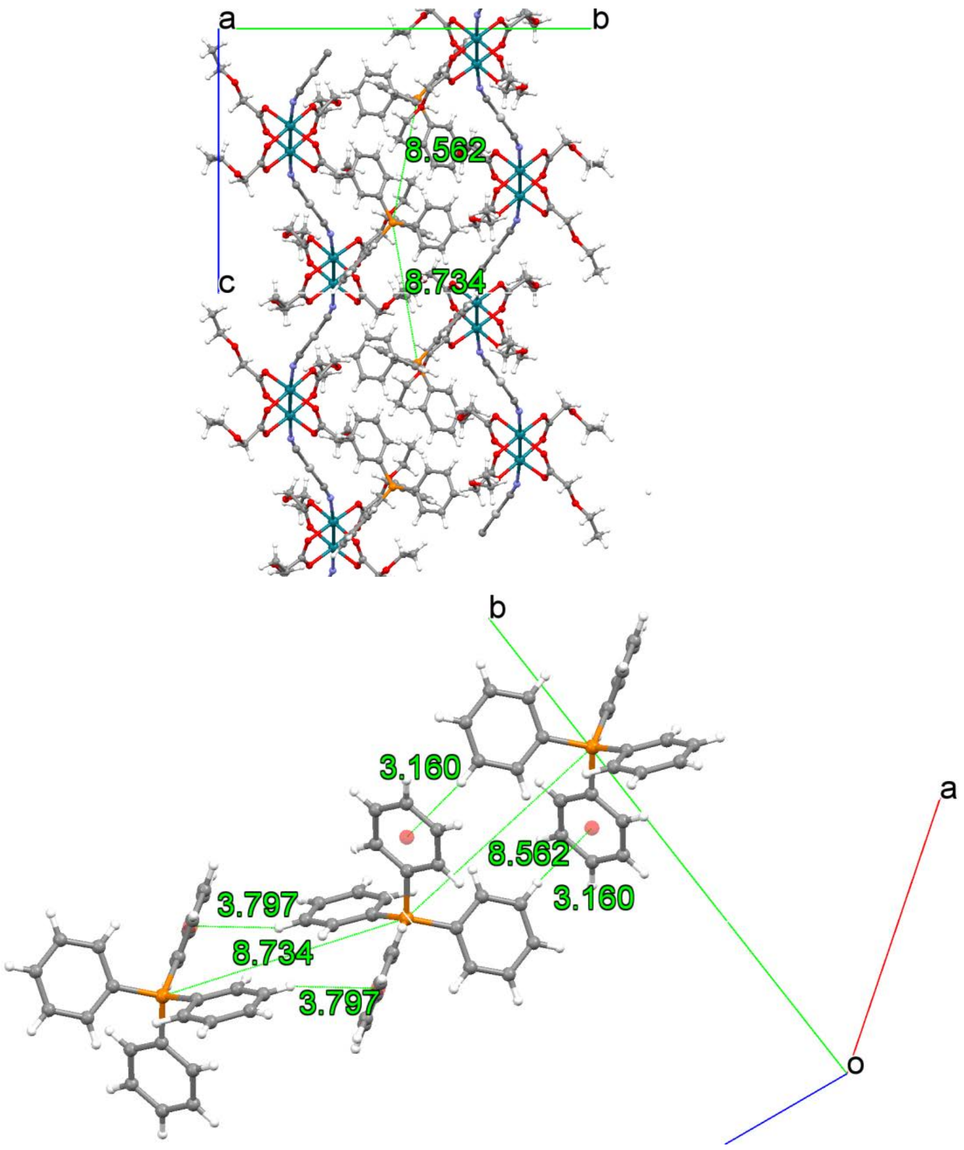

Figure S12. Interactions found between the closest $(PPh_4)^+$ cations in the structure of of **5**.

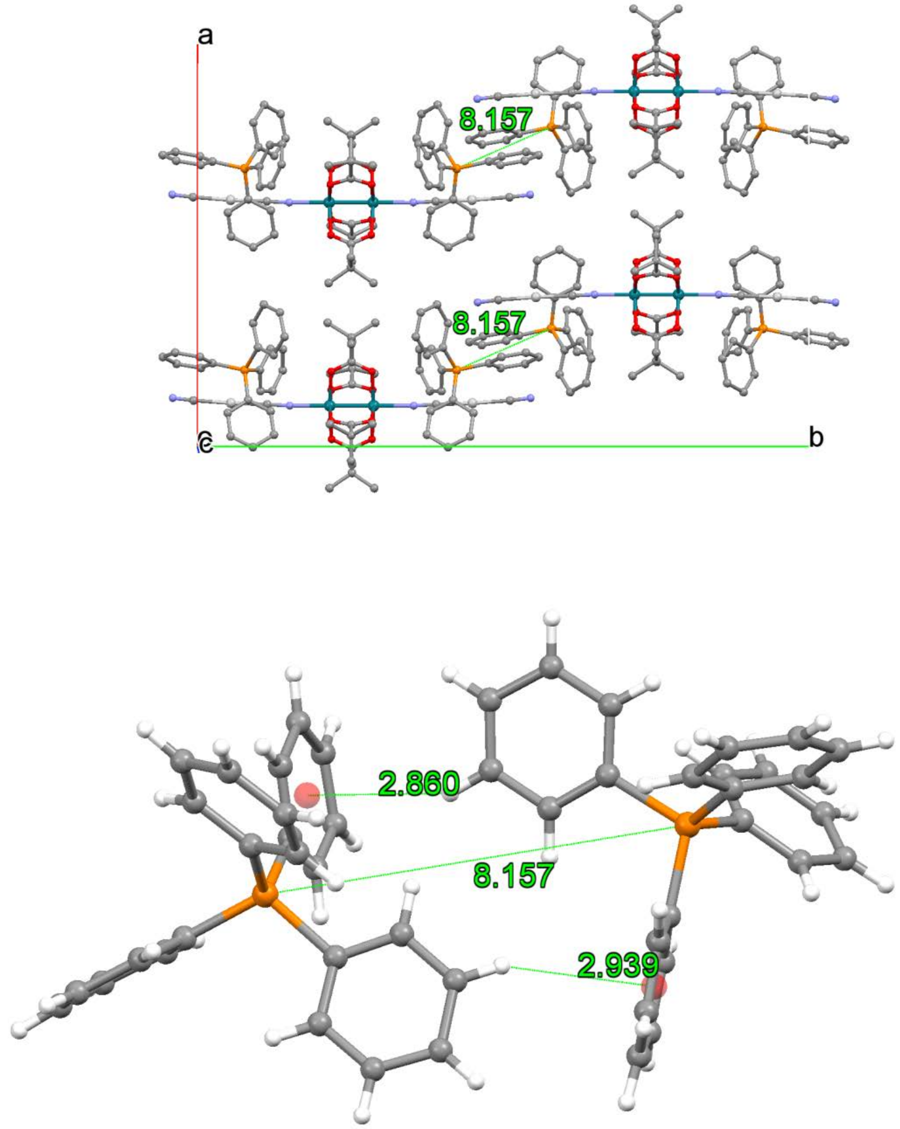

Figure S13. Representation of the structure of **6** along the c axis (top) and view of the interactions between the closest $(PPh_4)^+$ cations (bottom).

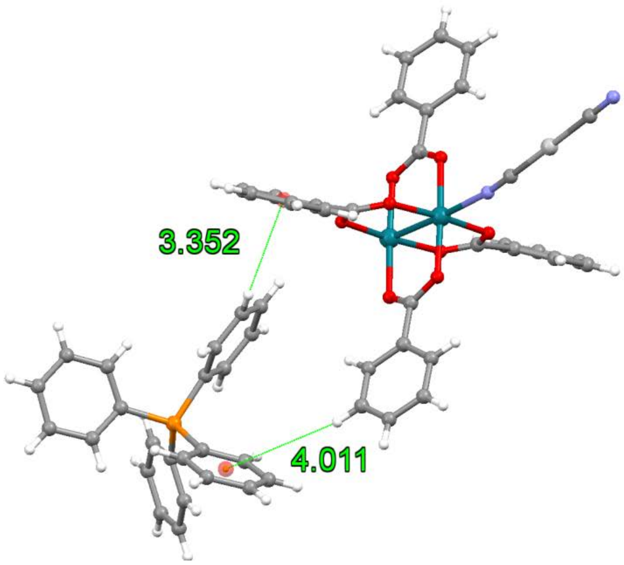

Figure S14. Interactions found between the closest cations and anionic chains in the structure of **4** .
